# Iliacus hematoma causing late femoral nerve palsy

**DOI:** 10.1002/ccr3.8654

**Published:** 2024-03-07

**Authors:** Fırat Al, Muhammed Köroglu, Hüseyin Utku Özdeş, Okan Aslantürk

**Affiliations:** ^1^ Department of Orthopedics and Traumatology Inonu University Faculty of Medicine Malatya Turkey; ^2^ Yesilyurt Hasan Çalık State Hospital Orthopedics and Traumatology Malatya Turkey

**Keywords:** decompression, femoral nerve, hematoma, iliacus muscle

## Abstract

Femoral nerve palsy is rare and may progress insidiously, leading to late clinical presentation. Identifying the underlying cause is very important for treatment. An iliacus hematoma causing nerve palsy is perhaps the most innocent etiology. However, this hematoma sometimes causes only abdominal pain and may even be misdiagnosed as a late intra‐abdominal pathology.

## Graphical abstract

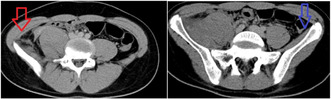

## 1 INTRODUCTION

Femoral nerve palsy due to iliopsoas hematoma is rare but has been previously defined in the literature. It is generally reported in case presentations as a complication of anticoagulant use.^1^, ^2^, ^3^ Hematoma has also been shown to develop after bleeding into the iliacus muscle in diseases with bleeding diathesis such as hemophilia.^4^, ^5^ The resulting hematoma affects the femoral nerve and causes hip and thigh pain, motor symptoms such as weakness in hip flexion and knee extension, and sensory symptoms such as hypoesthesia or anesthesia in the medial thigh. It may also give systemic findings such as decreased hematocrit, tachycardia, and hypotension secondary to bleeding.^6^ If the hematoma increases in size, it can be palpated like a mass and can cause abdominal pain.

In our case, we present a patient who applied to the emergency department with isolated abdominal pain mimicking appendicitis after a simple fall, and peripheral nerve findings appeared 25 days later. We have aimed to present a one‐year follow‐up of a case of femoral nerve palsy secondary to traumatic iliopsoas hematoma.

## 2 CASE HISTORY

A 15‐year‐old male patient was brought to the emergency department after falling from the same level while doing sports. He did not have any complaints other than abdominal pain at the time of his first admission. After blood tests and an outpatient abdominal roentgenogram performed in the emergency department, no pathology was detected, and the patient was discharged with non‐steroidal anti‐inflammatory drugs (NSAID) for pain. The patient had repeated emergency visits after complaints of abdominal pain that did not improve with NSAID treatment. An abdominal tomography evaluation was performed with a prediagnosis of appendicitis for right lower abdominal pain that increased 3 weeks after the initial presentation.

## 3 METHODS

Since the patient had repeated emergency admissions and abdominal pain was the only complaint, a prediagnosis of appendicitis was considered, and he was hospitalized in the pediatric surgery service. While the patient was being followed up in the pediatric surgery service, computed tomography (CT) was performed, and bleeding in the psoas muscle was detected and referred to us. There was no known disease and no history of drug use in the patient's previous medical history. The patient's vital signs were normal. Physical examination revealed an antalgic gait in the right lower extremity, which was detected by inspection. Significant atrophy was observed in the right thigh compared to the left side. In the supine position, the patients had active flexion of the trunk secondary to abdominal pain, and the knee was in a mild flexion position due to limitation in active extension. On palpation of the right lower quadrant of the abdomen, there was pain spreading to the right hip and thigh in the form of numbness. There was no patellar tendon reflex. There was loss of sensation in the medial and anterior right thigh, anterior femoral cutaneous nerve, and saphenous nerve dermatome. Manual muscle testing revealed right hip flexion with a motor power of 3/5 and right knee extension with a motor power of 2/5. X‐ray radiographs of the hip and knee showed normal osseous and articular tissues. Hematologic and biochemical values were normal in the blood tests of the patient. Bleeding diathesis due to factor deficiency was not detected.

**FIGURE 1:**
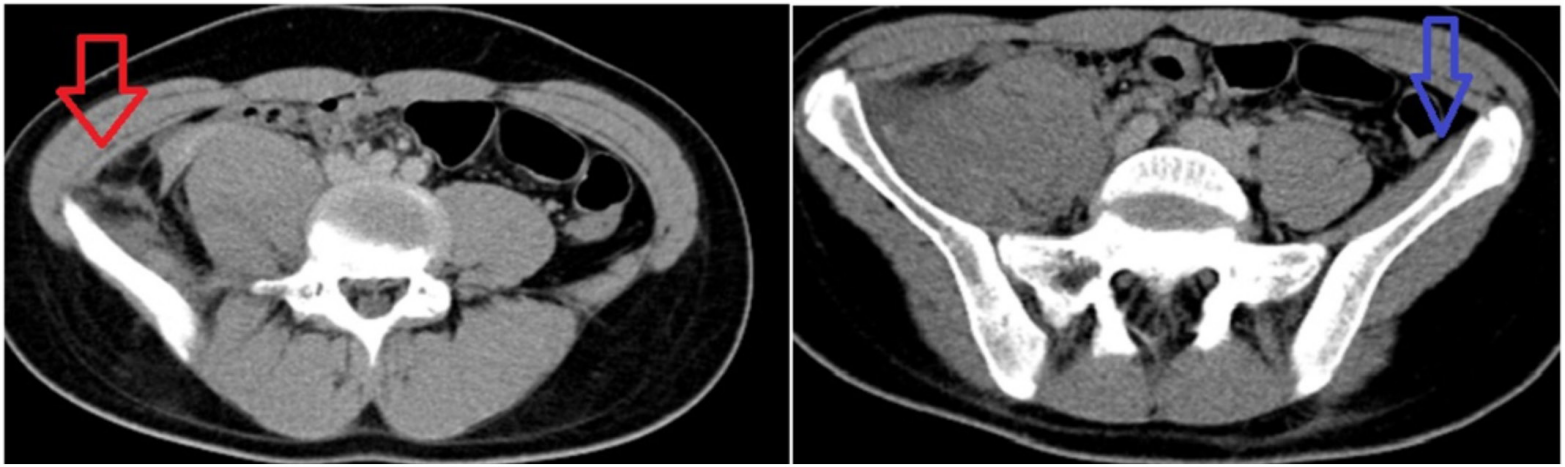
Hematoma causing femoral nerve palsy within the right iliacus muscle (red arrow), normal left iliacus muscle (blue arrow).

The patient underwent a CT examination to investigate the etiology of nerve paralysis, and electromyography (EMG) was performed to evaluate the femoral nerve condition in detail and to closely observe its progression. A contrast‐enhanced CT evaluation of the hip showed a hematoma in the right iliac muscle. (Figure 1). EMG evaluation revealed findings compatible with semi‐complete denervation of the right femoral nerve.

The patient was diagnosed with femoral nerve palsy secondary to iliopsoas hematoma, and surgery was planned for nerve decompression. Under general anesthesia, the fascia of the right iliac muscle was opened with an extraperitoneal approach to the right iliac fossa, and it was seen that the organized hematoma was compressing the nerve, and hematoma drainage and nerve decompression were achieved.

## 4 RESULTS

**FIGURE 2:**
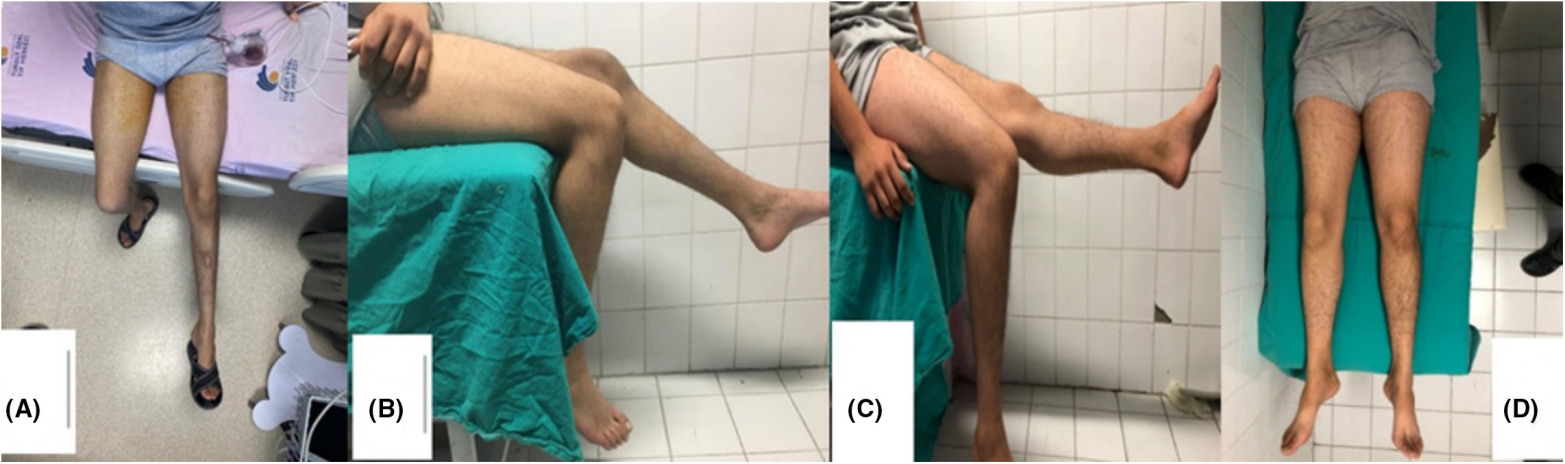
Preoperative thigh diameter difference (A), postoperative one‐month knee extension deficiency (B), and six‐month follow‐up full knee extension (C) and no thigh diameter difference (D).

In the postoperative 1st month follow‐up, hip flexor strength was 4/5 and knee extensor strength was 3/5; in the 6th month follow‐up, hip flexor strength and knee extensor strength were 5/5 (completely normal). The right thigh diameter difference was visibly improved at 6 months. One‐year follow‐up showed no signs of nerve damage (Figure 2).

## 5 DISCUSSION

The femoral nerve originates from the L2–L4 roots of the lumbar plexus, and during its course, it first branches to the psoas muscle and runs along the muscle. This nerve, whose anatomy is well known, has a route in the pelvis between the anteromedial aspect of the psoas muscle and the posterolateral aspect of the iliacus muscle. Femoral nerve pathologies have been associated with the neighborhood between the psoas and iliacus muscles during its anatomical course. Nobel et al.^7^ experimentally proved the possibility of compression neuropathy by injecting latex into the iliacus muscle fascia. In another study, it was shown that the blood formed after a tear in the iliopsoas muscle pressed on the femoral nerve, and nerve paralysis developed.^8^

The diagnosis of femoral nerve palsy is determined by a physical examination. On physical examination, motor weakness in hip flexion and knee extension, hypoesthesia or anesthesia in the anterior hip and medial thigh, and absence of patellar tendon reflex are diagnostic of femoral nerve palsy. EMG is guiding in the investigation of the etiology of the nerve abnormality, and it is often utilized in post‐treatment follow‐up.^9^ In addition to making a diagnosis, it is often more difficult to identify the underlying cause so that the right treatment can be provided. Nerve palsy is observed after direct trauma to the nerve, pelvic surgeries, the presence of a mass compressing the nerve, femoral artery catheterization, and graft harvesting from the iliac wing.^10^, ^11^, ^12^ Studies are reporting that femoral nerve palsy develops after a benign synovial cyst located near the nerve in this region, liposarcoma showing primary malignancy or metastatic features, and ewing sarcoma seen in the pediatric age group.^13^, ^14^, ^15^, ^16^ Our patient developed partial femoral nerve palsy with gradual progression secondary to hematoma after low‐energy trauma, and this condition developed in the subacute period.

The pathophysiology of iliacus hematoma has many controversies and is still under investigation. A rupture of the iliacus muscle has been implicated as a source of hemorrhage.^17^ Hemorrhage and hematoma formation after a pseudoaneurysm in the internal iliac artery have been shared.^18^ Another opinion is that hematoma develops after avulsion‐style tears in the small arteries supplying the iliacus muscle.^19^ Regardless of the source of bleeding, the main pathology in femoral nerve palsy secondary to hematoma formation is compression and stretching of the femoral nerve under the iliacus muscle fascia.

Case reports of femoral nerve palsy secondary to traumatic iliopsoas hematoma were shared in the literature. Kumar et al.^20^ reported a 20‐year‐old case of complete paralysis after blunt trauma. There are also case reports in which partial nerve damage developed and conservative treatment was applied.^21^, ^22^, ^23^, ^24^ There is a case of psoas hematoma in a patient who presented to the emergency department with spontaneous, isolated abdominal pain.^25^ In this study, findings of femoral nerve compression after acute hematoma were not observed, as in our study. This indicates that the hematoma has grown in time to the point of nerve paralysis. A common characteristic in studies with nerve deficits is the difference in the time it takes to develop nerve palsy and the time it takes to make the diagnosis, which is due to the timing of the onset of the patient's extremity complaints. In our case, after the original diagnosis was missed in the first stage, there were repeated emergency room admissions with complaints of persistent abdominal pain. This case illustrates the fact that the initial symptoms of iliopsoas hematoma clinic in the acute phase may mimic appendicitis findings and obscure the actual diagnosis.

There is no consensus on the treatment of nerve palsy secondary to iliopsoas hematoma. Partial nerve palsy may turn into complete paralysis with an increase in the size of the hematoma formed after trauma. On the contrary, a case in which no nerve palsy developed after hematoma development has also been published.^26^ In a case with complete nerve palsy treated conservatively, permanent sensory deficit was found in a one‐year follow‐up.^27^ In some studies presenting cases with partial nerve damage, the results of conservative treatment were reported as good, and complete recovery was demonstrated.^23^, ^24^, ^28^ In these studies, the case with the longest delay in diagnosis was presented as 25 days, and functional complete recovery was reported in the 6th month of follow‐up. In the literature, patients with traumatic iliopsoas hematoma with a total femoral nerve deficit were generally decompressed with surgical treatment, as we prefer.^17^, ^20^, ^29^ All of these studies presenting the results of complete nerve palsy cases showed complete recovery.

In light of these studies, it is seen that the success of surgical treatment in complete nerve damage is high. However, the management of partial nerve injuries seems to be a bit more sophisticated. Some specialists recommend both surgical and conservative treatment for partial nerve damage. In our case, there was a delay of 25 days. This long delay encouraged us for surgery. Conservative treatment can be tried in a patient presenting with partial nerve damage in the acute period, but we think that surgical decompression in the subacute period is effective in nerve recovery.

## 6 CONCLUSION

Femoral nerve palsy secondary to traumatic iliopsoas hematoma is rare and may progress insidiously. Physical examination, radiologic demonstration of the hematoma, and EMG, which shows the level of denervation of the nerve, help recognize cases of complete nerve palsy in the acute phase. The patient's history and nerve examination findings of the affected extremity lead us to the diagnosis, but in a case like ours, where abdominal pain is the only complaint at the beginning, iliopsoas hematoma may mimic other abdominal diseases until the femoral nerve findings appear, making it difficult to make the main diagnosis.

## AUTHOR CONTRIBUTIONS

**Fırat Al:** Conceptualization; investigation; methodology; writing – original draft; writing – review and editing. **Muhammed Köroglu:** Conceptualization; methodology; writing – original draft; writing – review and editing. **Hüseyin Utku Özdeş:** Conceptualization; data curation; formal analysis; funding acquisition; investigation; methodology; project administration; writing – original draft; writing – review and editing. **Okan Aslantürk:** Conceptualization; data curation; investigation; writing – review and editing.

## FUNDING INFORMATION

The authors received no financial support for the research and/or authorship of this article.

## CONFLICT OF INTEREST STATEMENT

The authors declared no conflicts of interest concerning the authorship and/or publication of this article. Funding: The authors received no financial support for the research and/or authorship of this article.

## CONSENT

A written informed consent was obtained from the patients.

## Data Availability

The data that support the findings of this study are available from the corresponding author upon reasonable request.
